# The Anticoagulation Dilemma: Concurrent Traumatic Brain Hemorrhage and Cerebral Venous Thrombosis in a Blast Injury Patient

**DOI:** 10.1002/ccr3.73458

**Published:** 2026-08-31

**Authors:** Ayenew Amare, Mikiyas G. Teferi, Laltu M. Negasa, Tesfaye A. Haile, Selamawit M. Bonsu, Kalkidan P. Ageze, Gedefaw T. Minwagaw

**Affiliations:** ^1^ Department of Emergency Medicine and Critical Care, School of Medicine, College of Health Sciences Addis Ababa University Addis Ababa Ethiopia; ^2^ School of Medicine, College of Health Sciences Addis Ababa University Addis Ababa Ethiopia

**Keywords:** anticoagulation, blast injury, case report, cerebral venous thrombosis, quadriparesis

## Abstract

Blast injuries produce complex multi‐system trauma through primary barotrauma, secondary penetrating projectiles, tertiary displacement forces, and quaternary mechanisms. Among the most challenging complications is the concurrent presentation of traumatic intracranial hemorrhage and cerebral venous sinus thrombosis (CVST), which creates a profound therapeutic dilemma regarding anticoagulation management. We present a 26‐year‐old male who sustained injuries from a high‐order explosive device that resulted in 19 fatalities. He presented 16 h post‐injury with a Glasgow Coma Scale score of 10/15 and marked quadriparesis with muscle power of 2/5 in all four limbs. Advanced neuroimaging ruled out cervical spine injury as the etiology of his motor deficits. Non‐contrast brain CT revealed a left posterior parietal comminuted and depressed skull fracture with multiple intracerebral bone fragments, associated hemorrhagic contusion, and perilesional edema. CT venography demonstrated extensive thrombosis of the superior sagittal sinus extending to the confluence of sinuses. Chest CT confirmed pulmonary contusions and subcutaneous emphysema. The patient received mannitol for cerebral edema management, phenytoin for seizure prophylaxis, and broad‐spectrum antibiotics. Following multidisciplinary consultation, therapeutic anticoagulation was withheld in the acute phase due to substantial risk of hemorrhage expansion from the adjacent contusion and penetrating bone fragments. The patient's neurological status stabilized over five days, though quadriparesis persisted at discharge. He was referred for neurosurgical evaluation and intensive rehabilitation, with anticoagulation initiated one week later. This case underscores that blast‐related TBI can produce severe central motor deficits without spinal injury, and that the coexistence of hemorrhagic contusion and CVST often contraindicates early anticoagulation, requiring individualized risk–benefit assessment and multidisciplinary decision‐making in severe neurotrauma.

**Key Clinical Message**

Blast‐related traumatic brain injury with concurrent hemorrhagic contusion and cerebral venous sinus thrombosis presents a profound therapeutic dilemma where withholding anticoagulation in the acute phase may be warranted to prevent hemorrhage expansion.

## 1 Introduction

Blast injuries represent a significant and complex challenge in emergency and critical care medicine, particularly in regions affected by conflict or terrorism. These injuries result from a combination of mechanisms, categorized as primary (barotrauma from the blast wave itself), secondary (penetrating trauma from projectiles), tertiary (displacement of the victim by the blast wind), and quaternary (all other injuries, such as burns and inhalations) [1]. The central nervous system is exceptionally vulnerable to these forces, with traumatic brain injury (TBI) emerging as a leading cause of mortality and long‐term disability among survivors [2].

The pathophysiology of blast‐related TBI is multifaceted. The primary blast wave can cause diffuse neuronal injury through mechanisms such as spalling, implosion, and shearing, disrupting crucial cortical and subcortical pathways [3]. Concurrently, penetrating debris such as shrapnel or bone fragments from secondary blast effects can cause focal injuries to critical structures like the motor cortex, leading to significant neurological deficits [4].

A particularly challenging complication in severe blast‐related TBI is the co‐occurrence of intracranial hemorrhage and cerebral venous sinus thrombosis (CVST). This creates a profound therapeutic dilemma [5]. The standard of care for CVST involves systemic anticoagulation to prevent thrombus propagation and potentially fatal complications like venous infarction [6]. However, this intervention carries a substantial risk of exacerbating intracranial bleeding when a hemorrhagic contusion is present, making the risk–benefit analysis extremely difficult, especially in the acute traumatic setting where evidence‐based guidelines are lacking [7].

This case report details the presentation and management of a young male blast victim who presented with this complex triad of findings: a penetrating hemorrhagic TBI, an extensive superior sagittal sinus thrombosis, and central quadriparesis. It highlights the critical importance of advanced neuroimaging for accurate diagnosis and explores the management dilemma that arises when two life‐threatening conditions require opposing treatment strategies.

## 2 Case History/Examination

A 26‐year‐old male was transported via ambulance to a tertiary emergency department 16 h after sustaining injuries from a high‐order explosive device in an open‐space blast that resulted in 19 fatalities. Pre‐hospital management at a referring hospital included tetanus toxoid, intravenous ceftriaxone, and primary closure of a left arm laceration.

On arrival, triage assessment was RED (critical) with a Triage Early Warning Score (TEWS) of 8. Vital signs were: Blood Pressure 106/53 mmHg, Heart Rate 123 bpm, Respiratory Rate 20/min, and SpO2 97% on room air. The primary survey revealed a patent airway, symmetrical chest expansion, and strong peripheral pulses. A blood‐soaked dressing was noted on the occiput. Neurologically, the Glasgow Coma Scale (GCS) was 10/15 (E2V4M4). A key finding on detailed examination was *quadriparesis*, with muscle power graded 2/5 in all four limbs.

## 3 Differential Diagnosis, Investigation and Treatment

**FIGURE 1:**
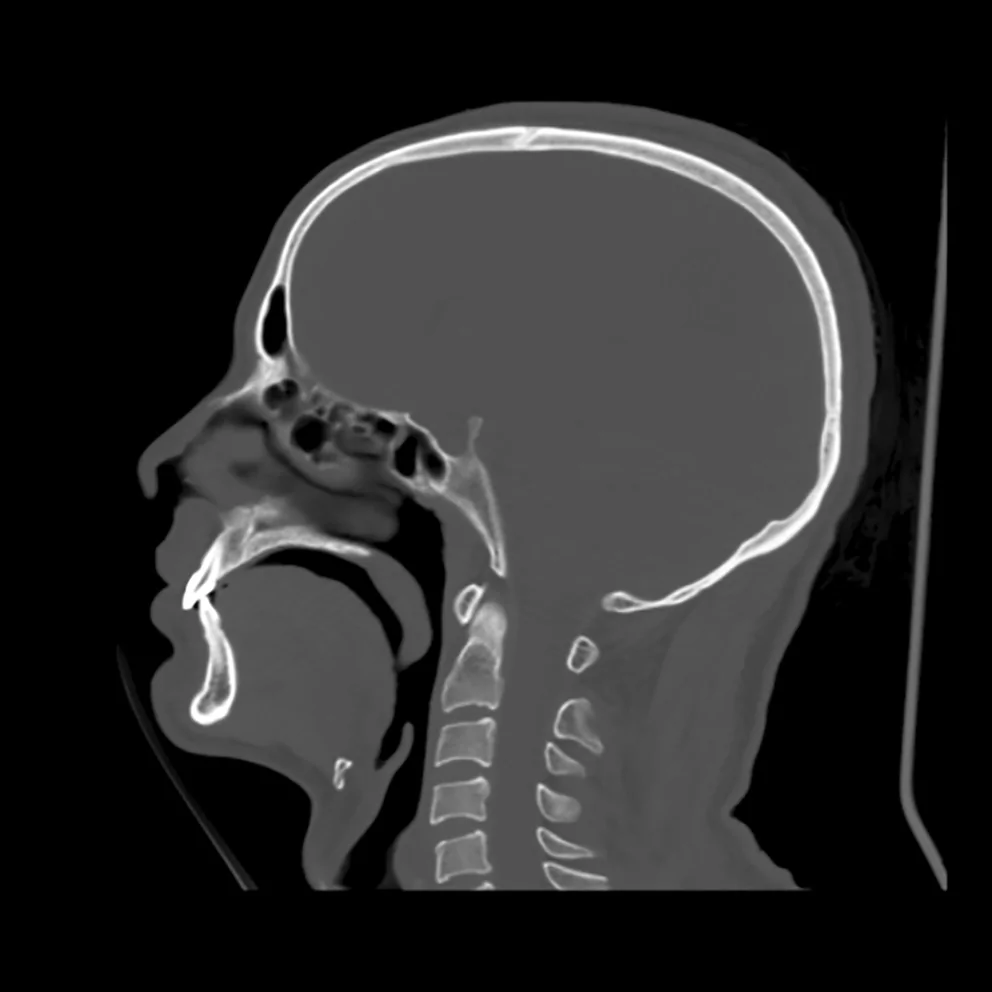
Non‐contrast sagittal CT images of the cervical spine demonstrating normal findings.

**FIGURE 2:**
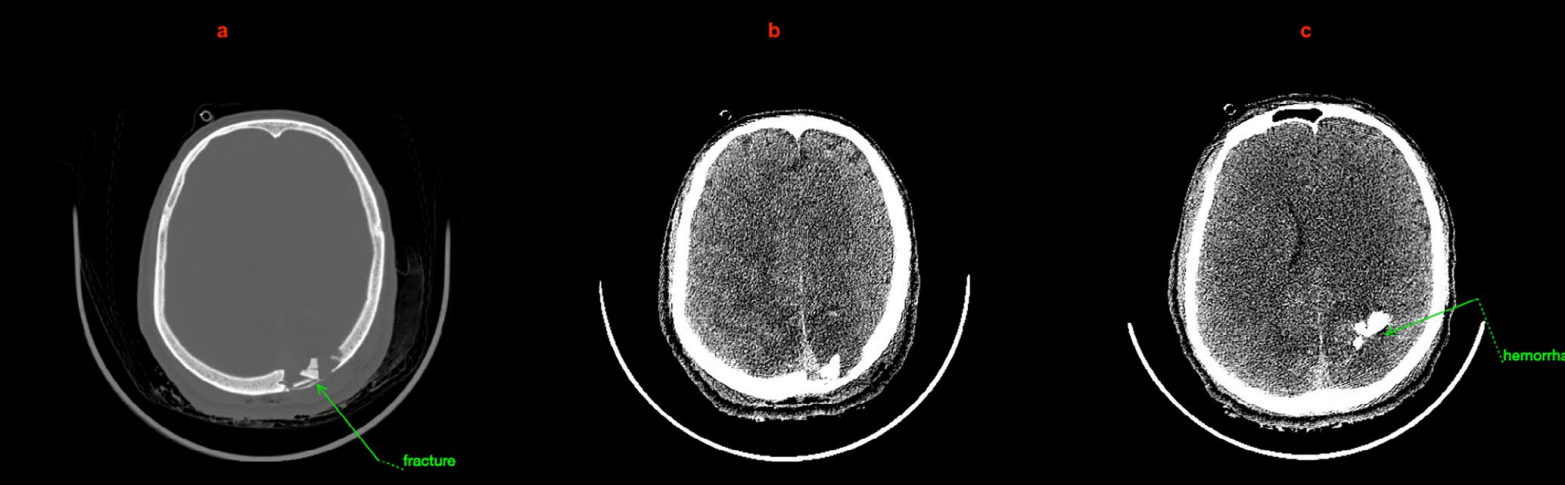
Axial brain CT images in bone (a) and brain (b & c) windows demonstrate a left posterior parietal comminuted and depressed calvarial fracture (green arrow in a) with multiple bone fragments lodged in the underlying brain parenchyma and intraparenchymal contusional hemorrhage and surrounding edema (green arrow in c).

**FIGURE 3:**
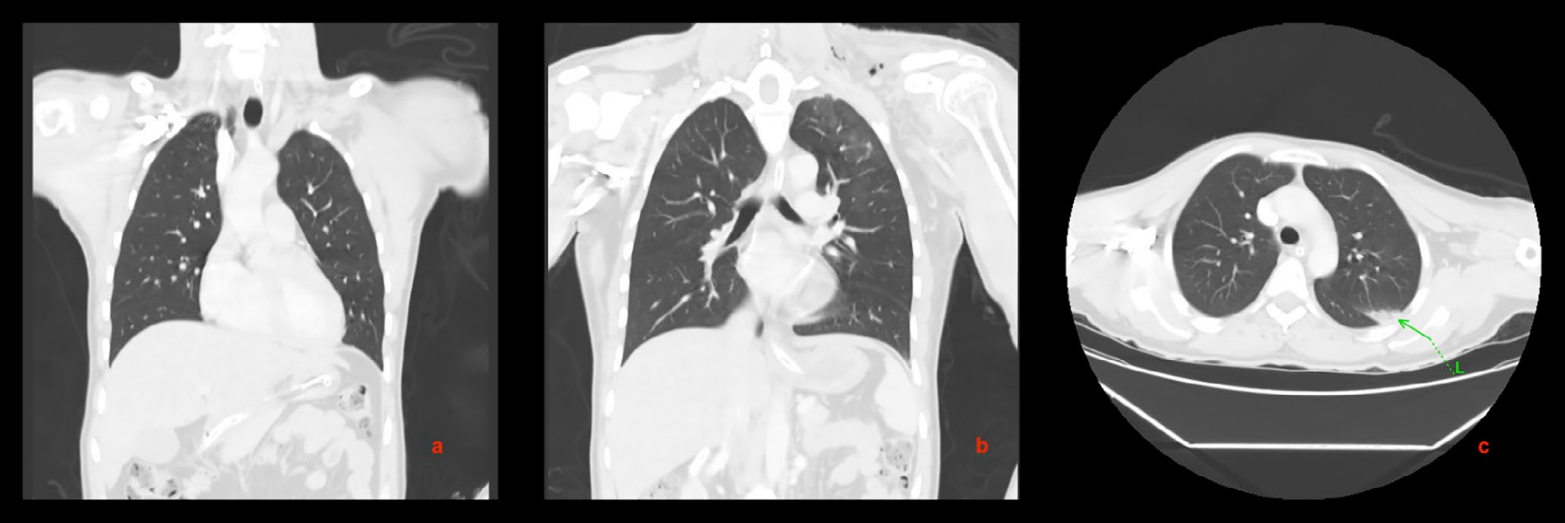
Sagittal & Axial chest CT image in lung window demonstrates nodular and coalescent ground‐glass opacities (GGOs) in the left upper lobe, consistent with pulmonary contusion and hemorrhage (arrow in c). Note is also made of left superficial neck subcutaneous emphysema.

**FIGURE 4:**
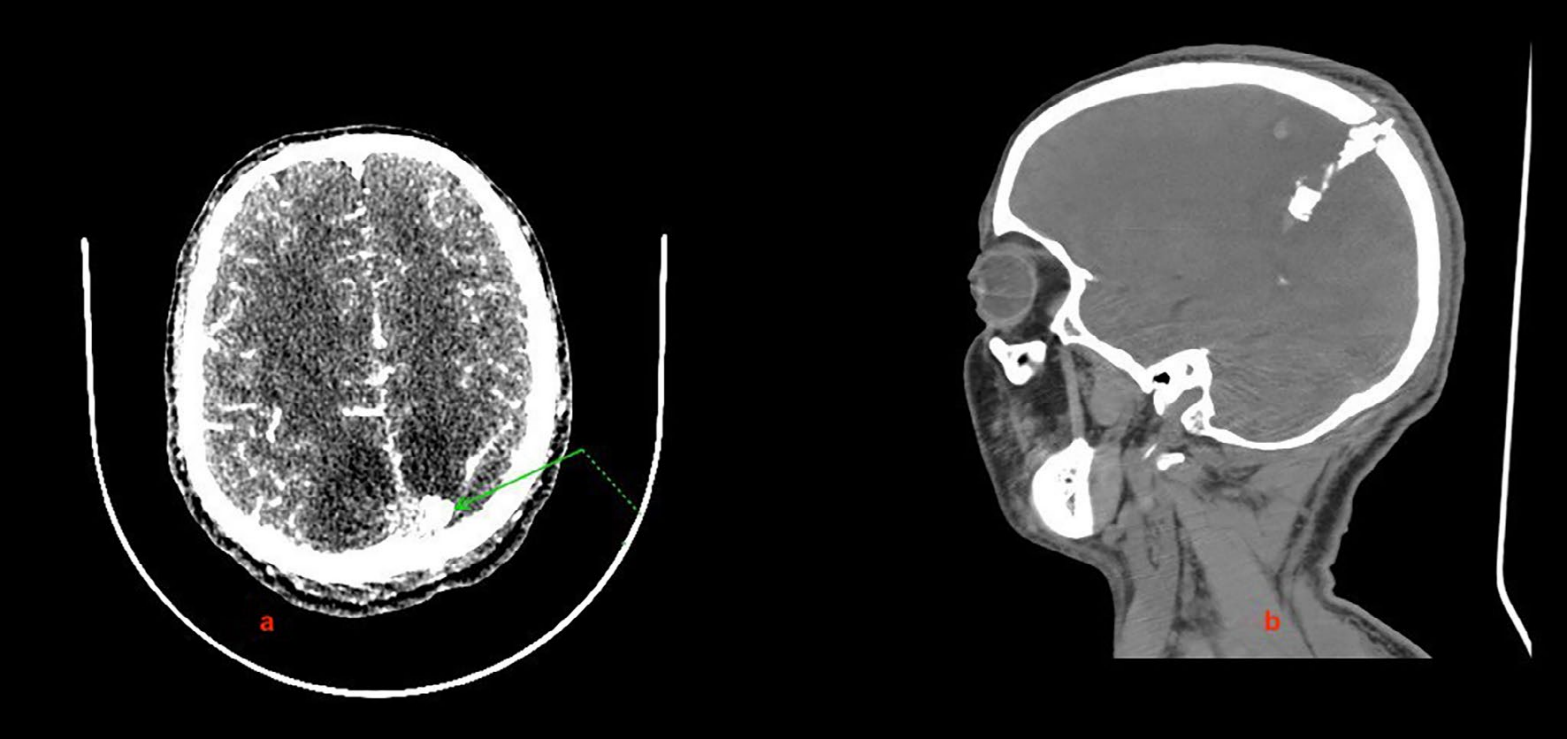
Contrast‐Enhanced Brain CT Venography. Axial (a) and sagittal (b) images from a CT venogram. The axial image represents contrast flow around a central thrombus. The sagittal image (b) confirms a long‐segment filling defect within the superior sagittal sinus extending to the confluence of sinuses.

Laboratory Investigations revealed a normal Complete Blood Count, Liver function test, Renal function test and electrolytes. Random Blood Sugar (RBS): 121 mg/dL and Blood was sent for group and crossmatch. Cervical Spine CT: No evidence of fracture, malalignment, or spinal canal compromise, effectively ruling out a spinal column injury as the cause of the quadriparesis (Figure 1). Non‐Contrast Brain CT revealed a left posterior parietal comminuted and depressed calvarial fracture with multiple bone fragments lodged in the underlying brain parenchyma. Associated intraparenchymal contusional hemorrhage and edema were noted along the path of injury (Figure 2a–c). The location of the injury in the parietal lobe, near the paracentral region, was consistent with the observed motor deficits. Chest CT demonstrated nodular and coalescent ground‐glass opacities (GGOs) in the left upper lobe, consistent with pulmonary contusion and hemorrhage. Left superficial neck subcutaneous emphysema was also present (Figure 3a–c). Contrast‐Enhanced Brain CT & CT Venography confirmed the above findings and revealed a critical additional finding: a long‐segment thrombosis of the superior sagittal sinus extending to the confluence of sinuses (Figure 4a,b).

## 4 Outcome and Follow‐Up

The patient was admitted with a diagnosis of moderate to severe TBI, comminuted‐depressed skull fracture with traumatic intracranial hemorrhage, cerebral venous sinus thrombosis (CVST), quadriparesis secondary to TBI, and blast lung. Initial management included neurosurgery consultation, Mannitol for cerebral edema, Phenytoin for seizure prophylaxis, and a course of antibiotics. Invasive intracranial pressure monitoring was not available. Neurological status was monitored through serial clinical examinations and repeat imaging when clinically indicated. Additional measures to mitigate intracranial hypertension included head elevation, maintenance of adequate oxygenation and hemodynamic stability, and administration of mannitol. A multidisciplinary team discussed the CVST but decided to withhold therapeutic anticoagulation due to the high risk of expanding the hemorrhagic contusion adjacent to the bone fragments. During this period, mechanical venous thromboembolism prophylaxis with intermittent pneumatic compression devices was employed to reduce the risk of systemic deep venous thrombosis while therapeutic anticoagulation remained contraindicated. The patient's condition stabilized over 5 days, though quadriparesis persisted. He was discharged with arrangements for intensive neurosurgical and rehabilitation follow‐up. At one‐week follow‐up, anticoagulation therapy was commenced with rivaroxaban.

## 5 Discussion

This case presents a remarkable illustration of the complex neurological sequelae that can follow blast exposure and the profound management challenges they pose. The initial finding of quadriparesis appropriately triggered concern for spinal column injury, a common assumption in polytrauma patients with motor deficits [7]. However, advanced neuroimaging definitively excluded cervical pathology and localized the cause of quadriparesis to a penetrating parietal lobe injury. This highlights a crucial teaching point: in blast‐related TBI, significant motor deficits can be solely of central origin, necessitating comprehensive neurological assessment and imaging [8].

The pathophysiological mechanisms underlying this patient's presentation are multifaceted. The primary blast wave likely caused diffuse axonal injury through acceleration–deceleration and shearing forces, while the penetrating bone fragments caused focal destruction of neural tissue in the parietal region [9]. The proximity of the injury to the paracentral lobule, which contains critical aspects of the primary motor and sensory cortices for contralateral limb function, provides a plausible anatomical explanation for the quadriparesis [10]. Additionally, the extensive superior sagittal sinus thrombosis likely contributed to global neurological dysfunction through impaired venous drainage, increased intracranial pressure, and potential venous ischemia [11].

The management of concomitant CVST and hemorrhagic contusion represents a significant therapeutic dilemma with limited evidence‐based guidance [12]. The American Heart Association/American Stroke Association guidelines suggest that anticoagulation may be considered for CVST even in the presence of intracerebral hemorrhage, as the hemorrhage is often venous in origin and may worsen from uncontrolled thrombosis [13]. However, these recommendations primarily address spontaneous CVST rather than trauma‐induced cases with active parenchymal bleeding and penetrating brain injury [14]. In trauma patients, the risk of hematoma expansion often takes precedence, particularly when the hemorrhage is large and associated with mass effect or unstable clinical status [15].

Our decision to withhold anticoagulation was based on several factors: the acute nature of the trauma, the presence of penetrating bone fragments within brain parenchyma, the significant hemorrhagic component adjacent to these fragments, and the patient's tenuous but stable neurological status. This conservative approach aligns with management principles in other cases of traumatic intracranial hemorrhage where the risk of rebleeding is substantial [16]. Instead, we opted for close neurological monitoring and serial imaging to detect any early signs of thrombotic progression or hemorrhagic expansion.

The management of intracranial pressure also warrants consideration. Although Brain Trauma Foundation guidelines recommend invasive ICP monitoring in selected patients with severe traumatic brain injury, such monitoring was not available in our setting [6,14]. Consequently, management relied on serial neurological examinations, neuroimaging surveillance, osmotherapy with mannitol, head elevation, and optimization of physiological parameters. Similarly, mechanical VTE prophylaxis was utilized while anticoagulation was deferred, balancing the competing risks of thrombosis and hemorrhagic progression.

The persistence of quadriparesis at discharge underscores the severe nature of this patient's brain injury. Motor recovery following such injuries is often incomplete and requires intensive, prolonged rehabilitation [17]. The prognosis for significant functional improvement depends on multiple factors including the extent of initial neural damage, the effectiveness of rehabilitation interventions, and the development of complications such as post‐traumatic epilepsy [18].

This case also highlights the critical importance of blast lung screening in patients with explosive injuries. While our patient's respiratory status was initially stable, the CT findings of pulmonary contusions and subcutaneous emphysema confirmed significant thoracic barotrauma that required monitoring for potential respiratory deterioration [19].

## 6 Conclusion

This case underscores several critical principles in managing complex blast injuries. First, profound motor deficits like quadriparesis can originate directly from traumatic brain injury, even without spinal column damage, necessitating thorough neuroimaging. Second, the concurrence of hemorrhagic brain injury and cerebral venous thrombosis creates a formidable therapeutic dilemma, where the risk of anticoagulation often outweighs its benefits in the acute phase, prioritizing damage control and neurological stabilization. Finally, optimal outcomes for such patients hinge on a multidisciplinary approach, advanced imaging, and individualized decision‐making in the absence of definitive guidelines. This report highlights the urgent need for further research to establish protocols for managing these competing pathologies in severe neurotrauma. It also underscores the necessity for developing structured protocols, potentially involving serial imaging and thrombophilia testing, to guide the timing of anticoagulation in similar high‐risk scenarios.

## Author Contributions

**Ayenew Amare:** project administration, resources. **Mikiyas G. Teferi:** writing – original draft. **Laltu M. Negasa:** validation, visualization. **Tesfaye A. Haile:** resources. **Selamawit M. Bonsu:** investigation. **Kalkidan P. Ageze:** software. **Gedefaw T. Minwagaw:** supervision.

## Funding

The authors have nothing to report.

## Ethics Statement

The authors have nothing to report.

## Consent

Written informed consent was obtained from the patient for the publication of personal and clinical details, including any identifying images.

## Conflicts of Interest

The authors declare no conflicts of interest.

## Data Availability

The data supporting the findings of this case report are available from the corresponding author upon reasonable request.
